# Leadership avoidance as a rational choice among women instructors in higher education: cross-cultural parallels

**DOI:** 10.3389/fpsyg.2026.1767636

**Published:** 2026-04-10

**Authors:** Benazir Ayesha, Bo Wen, Yuan Li, Fawad Ahmed

**Affiliations:** 1 Xichang University, Xichang, China; 2 Swansea University, Swansea, United Kingdom; 3 Xi'an Jiaotong-Liverpool University, Suzhou, China

**Keywords:** coping-capital legibility, leadership avoidance, role stress, role-congruity climate, women academics

## Abstract

**Introduction:**

Women’s reluctance to pursue formal leadership in higher education is often framed as an individual deficit rather than as a response to institutional conditions. This study reconceptualises leadership avoidance as a rational choice arising from gendered contexts that generate role stress, personal strain, and leader identity threat. It examines whether these mechanisms operate similarly across two contrasting higher education systems and whether individual and institutional resources weaken this pathway.

**Methods:**

The study tests a mediated model linking role stress to leadership avoidance through personal strain and leader identity threat. Data were collected from matched samples of women academics in the United Kingdom (*n* = 236) and Pakistan (*n* = 229). Structural equation modelling was used to assess the proposed relationships and to examine the moderating roles of coping-capital legibility, role-congruity climate, and leader identity.

**Results:**

The findings show that chronic role stress increases both personal strain and leader identity threat, which in turn heighten leadership avoidance. This indirect pathway is stronger in the United Kingdom’s high-performance, audit-driven environment than in Pakistan’s more relationally buffered but patriarchal context. High coping-capital legibility and strong leader identity reduce the strength of this pathway.

**Discussion:**

The results suggest that leadership avoidance among women instructors is better understood as a contextually rational response to gendered institutional demands than as a failure of ambition. By extending conservation of resources theory, the study introduces coping-capital legibility as a mechanism linking individual coping resources to institutional systems of recognition. The findings highlight the importance of recognition structures and identity-supportive climates in reducing leadership avoidance across higher education contexts.

## 1 Introduction

Existing literature attributes women’s under-representation in formal leadership roles to the lack of aspiration, ambition, or confidence (Schnackenberg, 2026; Madsen and Longman, 2020). However, a growing body of research reframes this notion, suggesting that leadership avoidance is not merely a result of disengagement but rather an adaptive response to institutional conditions that render leadership roles costly, precarious, or unrewarding (O’Connor, 2014). We conceptualise leadership avoidance as a resource-conserving and identity-protective strategy which emerges when chronic work-related demands erode personal and social resources and when organisational systems penalise or ignore leadership contributions (Colligan and Higgins, 2006; Babcock et al., 2017).

This line of thought suggests avoidance from leadership roles is not necessarily a sign of low motivation, but can be an adaptive, strategic decision under persistent structural and cultural barriers (O’Connor, 2014; Dominici et al., 2009) that make leadership costly and risky for women. Contemporary reviews and empirical work continue to document durable barriers in higher education, including gendered evaluation, informal exclusion, microaggressions, and the undervaluation of service and relational labour, even in systems with formal equality policies (Avolio et al., 2024; Fauzi et al., 2024; Harris et al., 2024; Meza-Mejia et al., 2023; Tang and Horta, 2024; Yahya et al., 2024). Accordingly, leadership avoidance is better framed as resource-conserving and identity-protective under institutional conditions that amplify the personal costs of leadership.

Conservation of Resources (COR) theory (Hobfoll, 1989, 2018) says people withdraw from roles and actions when resources are getting depleted. We build on this by showing that avoidance becomes more likely when institutions do not recognise coping efforts and when leadership climates create identity risk by embedding it in gendered organisational ecologies. This reframing moves beyond individual-level stress spirals to theorize organisational conditions that make leadership pursuit either rational or irrational, depending on the resources available and the recognition systems in place (O’Connor, 2014).

Despite extensive scholarship documenting women’s under-representation in higher education leadership, the literature remains more developed in identifying barriers than in explaining how those barriers translate into leadership avoidance. Islam et al. (2023), for example, call for further context-specific research on individual, socio-cultural, and organisational barriers, but do not explicitly model how such barriers interact to produce withdrawal from leadership. More broadly, systematic reviews and syntheses consistently show that women face persistent gendered constraints across higher education systems, including biased evaluation, disproportionate service burdens, exclusion from informal networks, and work-family penalties (Avolio et al., 2024; Harris et al., 2024; Meza-Mejia et al., 2023; Yahya et al., 2024). Yet much of this literature remains stronger at cataloguing obstacles than at specifying the psychological transmission through which institutional demands culminate in leadership avoidance (Schnackenberg, 2026). This omission matters because leadership avoidance cannot be inferred simply from the presence of barriers. What requires explanation is how role stress is translated into a withdrawal response.

This study addresses that omission by reframing leadership avoidance as a staged and theoretically ordered process rather than a diffuse outcome of disadvantage. Existing qualitative and review-based research offers valuable insight into how gender segregation, marginalisation, administrative overload, biased hiring, harassment, and work-personal conflict constrain women’s careers (Bowen, 2024; Hakiem, 2023; Harris et al., 2024; Yahya et al., 2024). However, these studies typically remain descriptive, correlational, or thematic, and therefore do not test whether role stress first intensifies personal strain through resource depletion, whether strain then heightens leader identity threat, and whether this combination ultimately makes leadership avoidance more likely. This sequential path represents a substantive theoretical advance because it differentiates analytically between strain and identity threat, specifies their ordering, and explains why women may avoid leadership not because of deficient aspiration, but because withdrawal becomes a rational resource-conserving response under sustained institutional pressure.

Reviews consistently note that women academics undertake substantial relational and often invisible labour, including mentoring, emotional regulation, and coordination, yet these contributions are frequently undervalued within promotion and leadership systems (Avolio et al., 2024; Harris et al., 2024; Yahya et al., 2024). The issue, then, is not simply that women cope, but whether coping work is institutionally recognised and convertible into valued returns. This is the conceptual space captured by coping-capital legibility, defined here as the perceived degree to which coping efforts are recognised, credited, and translated into institutional returns such as workload relief, sponsorship, or time flexibility. In this study, coping-capital legibility is theorised as a contextual moderator of the relationship between role stress and personal strain. The core idea is that role stress does not translate into strain with equal force under all institutional conditions. Rather, that translation depends partly on whether the coping work women perform, such as mentoring, relational coordination, and emotional support, is visible, credited, and convertible into valued organisational returns. When such coping labour is institutionally legible, it is more likely to generate replenishing resources, including recognition, workload accommodation, sponsorship, or greater discretion over time and support. Under those conditions, the positive association between role stress and personal strain should be weaker because coping efforts are more likely to offset rather than compound resource depletion. By contrast, when coping labour remains institutionally illegible, the same effort consumes time and emotional energy without producing meaningful return, making the stress to strain relationship stronger. This is consistent with research showing that women’s relational and pastoral labour in universities is frequently expected yet undervalued, such that its protective potential depends heavily on whether institutions actually recognise and reward it (Acker, 2012; Babcock et al., 2017; Husu, 2022; Quickfall, 2025). It is also consistent with evidence that recognised social support and coping resources can buffer strain, whereas unrecognised and gendered support work can intensify exhaustion and reduce resilience over time (Doyle and Hind, 2002; Kirmani et al., 2024; Lodhi et al., 2023; Van Emmerik, 2002).

According to this logic, it is theoretically appropriate to test phenomenon across contexts. Prior cross-national work on women’s academic leadership often relies on narrative comparison of country differences, which can generate useful descriptive insight but remains limited for theory testing (Harris et al., 2024; Meza-Mejia et al., 2023). More importantly, cross-context variation is theoretically expected because institutional architectures differ in ways that shape the recognisability of coping, the legitimacy of women’s leadership claims, and the pressures attached to leadership itself. For instance, evidence from China shows how merit-based fairness narratives can reproduce gender blindness and dampen support for gender-responsive interventions, thereby influencing whether women’s coping efforts are rewarded or dismissed (Tang and Horta, 2024). More generally, differences in performance regimes, meritocratic norms, and gendered role expectations are likely to shape the operation of the same underlying mechanism in predictable ways.

A review of the literature on women leadership in academia indicates there is a progressively layered omission. Prior research shows that women encounter barriers to leadership, but is less precise about when those barriers become psychologically depleting, when coping efforts fail to generate institutional return, and why these processes should intensify or weaken across contexts. Addressing that layered omission allows the present study to anchor itself more firmly in the leadership avoidance literature while also speaking to the broader question of women’s under-representation in higher education leadership (O’Connor, 2014).

To address these gaps, this paper compares two distinct higher-education systems: the UK and Pakistan, how psychological mechanisms and context-specific institutional factors are related to women’s leadership avoidance, and in the process advance COR Theory by observing how institutional recognition is linked with resource depletion and leadership avoidance. Accordingly, we examine whether role stress predicts leadership avoidance through personal strain and leader identity threat, and whether this pathway is conditioned by coping-capital legibility and role-congruity climate. Unlike constructs such as perceived organisational support or fairness of workload allocation, which focus on general support systems, coping-capital legibility emphasizes the conversion of coping behaviors into replenishing capital, thereby sustaining leader identity (DeRue and Ashford, 2010). This meso-level construct links micro-level coping behaviors to broader institutional evaluation systems, clarifying when coping stabilizes or undermines leadership trajectories.

In terms of contribution, this study first reconceptualises leadership avoidance as a rational, adaptive strategy rather than a deficit. By advancing theoretical clarity, it explains when capable professionals opt for withdrawal as a protective response to chronic resource depletion and identity threats (O’Connor, 2014). Offering a comparative, context-sensitive test of these mechanisms across two national higher-education systems, we analyse Pakistan as a “strong context” where gendered constraints amplify institutional dynamics; and compare it with the UK’s policy-rich but structurally biased academic environment. We identify both generalisable and context-specific pathways of leadership avoidance, which advances the debate from under-representation of women to “why leadership pursuit becomes rationally unsuitable”. This offers a theoretically grounded and actionable explanation of gendered leadership outcomes across academia (Acker, 2012; Quickfall, 2025). The study goes beyond descriptive comparisons to empirically test psychological mechanisms such as resource depletion and identity threat across different national systems. Using multi-group structural equation modeling, it rigorously compares the stress–strain-identity-threat mechanisms in the UK and Pakistan, enabling a more robust evaluation of theoretical universality and contextual contingency (Ghundol and Muthanna, 2025). Second, the study introduces and operationalises coping-capital legibility as a novel moderator, explaining how coping practices either mitigate or exacerbate resource depletion. This contributes to bridging individual coping literature and institutional evaluation theory (DeRue and Ashford, 2010). Third, from a methodological perspective, the paper advances existing research by integrating matched samples across two national contexts and testing moderated sequential mediation using structural equation modeling. This approach enhances empirical rigor and provides more predictive clarity in existing leadership-avoidance frameworks. Finally, the study extends COR theory into gendered academic ecologies, demonstrating how resource loss leads to identity threat only when coping behaviors lack institutional legibility, which culminates in withdrawal in masculinised leadership climates.

## 2 Theoretical background and hypotheses development

### 2.1 1 Conceptual foundations and construct specification

The theoretical lens herein integrates two established but intersecting literatures: occupational stress and gendered leadership. Occupational stress research demonstrates that sustained demanding work, such as long hours, emotional labor, and role overload, deplete psychological, temporal, and emotional resources, propelling individuals toward protective withdrawal when replenishment is uncertain (Colligan and Higgins, 2006; Kelly et al., 2011). In parallel, gender and leadership research has shown that women’s agentic behaviors are often not recognised as legitimate leadership, rendering their leadership aspirations riskier and more fragile compared to men’s (Eagly and Carli, 2007; Carnes et al., 2015). The intersection of these two factors of resource depletion and gendered organisational climates (Schnackenberg, 2026) channels women’s coping strategies away from visible leadership engagement towards strategic withdrawal (Babcock et al., 2017). Importantly, this withdrawal is not a manifestation of burnout or apathy, but a response to the institutional economies of recognition and risk (O’Connor, 2014). The table below introduces conceptual definitions and construct architecture of the study (see Table 1).

**Table 1:** Conceptual definitions and construct architecture.

Construct	Conceptual definition (this study)	Level	Model role
Role stress	The structural demands embedded in academic roles (e.g., overload, ambiguity, conflict) that require sustained effort and create pressure to perform. In this study, job demands are operationalised via role stress.	Individual perception of structural demands	Exogenous predictor
Personal Strain	The psychological depletion response to sustained role stress, reflecting reduced emotional and cognitive capacity and fatigue. Strain is treated as the *manifestation* of resource depletion, not a synonym for stress.	Individual psychological state	Mediator 1
Leader identity threat	Perceived risk to one’s legitimacy, safety, or acceptance when enacting leadership in contexts where leadership prototypes are gendered and women face evaluative scrutiny. This is socially evaluative and distinct from depletion.	Individual identity appraisal	Mediator 2
Coping-capital legibility	The perceived degree to which coping labour (e.g., mentoring, relational coordination, emotional labour) is visible, credited, and convertible into valued institutional capital (e.g., sponsorship, workload relief, career credit).	Meso-level recognition signal (perceived)	Moderator of stress–strain
Role-congruity climate	The perceived climate signalling whether leadership is masculinised and whether women incur penalties for authority and visibility.	Meso-level climate (perceived)	Moderator of identity threat—leadership avoidance
Leadership avoidance	Reluctance to apply for, accept, or sustain formal leadership roles because anticipated costs exceed expected returns, expressed behaviourally and cognitively.	Behavioural intention/withdrawal	Dependent variable

To avoid construct drift, we specify the model architecture explicitly. The exogenous predictor is roles stress. The model contains two ordered mediators: personal strain followed by leader identity threat. The outcome is Leadership Avoidance. Two contextual moderators are specified: coping-capital legibility moderates the role stress—personal strain path by capturing perceived institutional convertibility of coping work into valued capital (that is, whether mentoring and emotional labour yield tangible relief or recognition). Role-congruity climate moderates the leader identity threat to leadership avoidance path by capturing the perceived masculinisation of leadership and the penalty risk for women who enact authority (Eagly and Karau, 2002; Hobfoll, 1989, 2018).

### 2.2 Leadership avoidance

**Figure 1:**
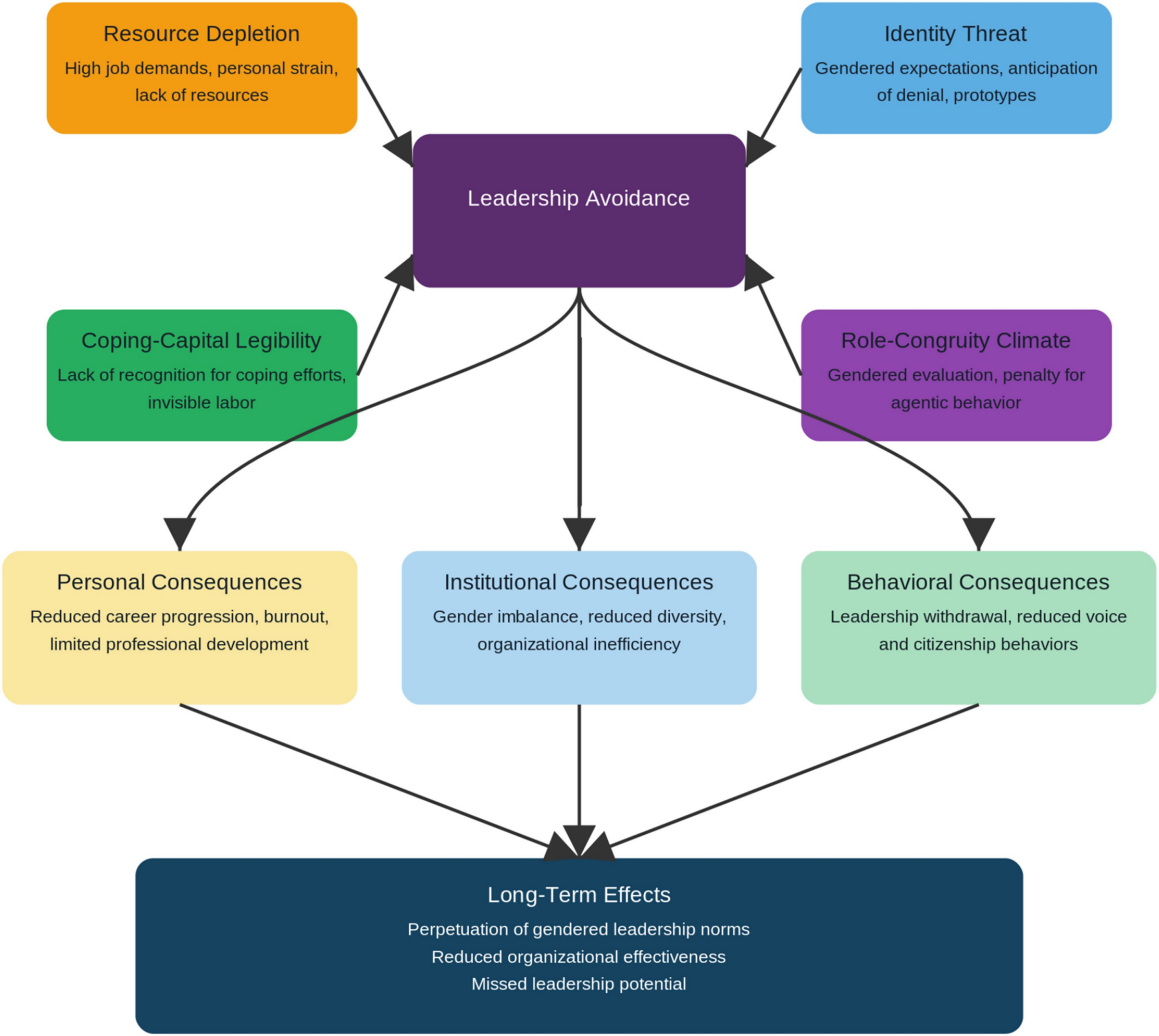
Antecedents and consequences of leadership avoidance. Source: Authors’ own construction adapted from and based on works of Ayyildiz and Banoglu (2024), Burkinshaw and White (2017), Eagly and Karau (2002), Ghundol and Muthanna (2025), Schnackenberg (2026), Quickfall (2025), Bhatti and Ali (2021), and Asrifan et al. (2026).

Understanding leadership avoidance among female academics requires integrating multiple theoretical perspectives explaining how gendered institutional structures create leadership gaps (Diehl and Dzubinski, 2016). We focus on chronic strain, identity threat, and withdrawal from leadership roles through three complementary theories offering a comprehensive explanation (see Figure 1).

Firstly, Role Theory (Kahn et al., 1964; Rizzo et al., 1970) posits that stress arises when individuals face conflicting expectations, excessive demands, or unclear role boundaries. Female academics navigate multiple roles of lecturer, researcher, mentor, caregiver and each with distinct pressures. The overlap between professional and domestic roles creates persistent role overload and ambiguity, undermining well-being and engagement. Leadership avoidance becomes a rational outcome under excessive and conflicting demands. Second, Conservation of Resources (COR) Theory (Hobfoll, 1989) extends this by emphasizing that individuals aim to acquire, protect, and maintain valued resources such as time, autonomy, and status. When resources are depleted without replenishment, individuals adopt protective strategies. For female academics, leadership roles increase workload and visibility, further depleting resources. Therefore, opting out of leadership is a resource-conservation strategy, not a sign of low aspiration. COR theory also predicts a cumulative depletion spiral, where unreciprocated efforts (e.g., unpaid mentoring) deepen resource loss and reinforce avoidance. Third, Role-Congruity Theory (Eagly and Karau, 2002) links stress and resource loss to identity threat. Leadership is viewed as a masculine domain emphasizing agency, while women are expected to be communal. This incongruence produces double-bind pressures: women who act agentically face backlash, while those who act communally are perceived as weak. Consequently, female academics internalise leadership as a high-cost, low-reward endeavor, where deviation from gender norms invites skepticism and sanction. These theories intersect in explaining leadership avoidance as a rational strategy for protecting identity and resources (DeRue and Ashford, 2010). Female academics’ reluctance to pursue leadership arises from structural and psychological asymmetries that make leadership riskier for women. Institutional contexts prioritizing masculine leadership models, devaluing relational labor, and imposing excessive service expectations exacerbate this imbalance (Acker, 2012; O’Connor, 2014).

Classic role stressors (overload, conflict, ambiguity) are structural antecedents of strain (Kahn et al., 1964; Rizzo et al., 1970). Among academics, these are intensified by gendered service expectations and undervalued boundary-spanning work (Guarino and Borden, 2017; Williams et al., 2023). In environments dominated by masculine leadership models, women anticipate lower recognition for leadership claims, framing strain as an identity threat rather than workload fatigue (Carnes et al., 2015). We integrate these insights through coping-capital legibility, defined as the degree to which institutions recognize and reward coping practices. When coping efforts are visible, they generate tangible returns such as recognition, time relief, or promotion, reducing stress and strain. When invisible, these efforts exacerbate resource loss and identity threat (Kelly et al., 2011).

### 2.3 Contrasting contexts from developed and underdeveloped countries

Testing these mechanisms across contexts matters for two reasons. First, it is a direct test of theory transportability. Conservation of resources (COR) theory predicts that sustained demands trigger depletion and leadership avoidance, but the strength and sequencing of these links can be contingent on institutional conditions that determine whether resources are replenished or further eroded (Hobfoll, 1989, 2018). Multi-group mechanism tests therefore show whether depletion dynamics are general psychological processes or whether they are structurally activated by specific institutional designs. Second, it improves intervention precision. If the stress—strain—threat—avoidance pathway is stronger in one system, then leadership programmes that focus only on “confidence” will misfire because they ignore the institutional amplifier. Cross-context mechanism testing allows universities to target the right lever, for example reducing demand exposure, increasing convertibility of support work into career capital, or dampening role-congruity penalties that make leadership identity-threatening (Harris et al., 2024; Tang and Horta, 2024).

We look at common psychological processes, and also see how institutional conditions in each country shape these processes. The UK has formal equality legislation and advanced human resource infrastructure. It is a high-resource context where gender inequities persist subtly through glass-ceiling effects, exclusion from networks, and undervalued emotional labor (Ellinas et al., 2018; Madsen and Longman, 2020). By contrast, Pakistan offers a low-resource, high-constraint context characterised by overt patriarchal hierarchies and opaque advancement systems (Ghundol and Muthanna, 2025). Examining both contexts allows us to identify universal psychological mechanisms such as resource loss, identity threat, and coping, as well as context-contingent institutional amplifiers, such as the legibility of coping and role-congruity climates (Acker, 2012; Husu, 2022).

In Pakistan’s public universities, institutional inequities, patriarchal hierarchies, opaque advancement criteria, and exclusionary male-dominated networks depress coping-capital legibility and amplify role-congruity pressures (Shah, 2015; Morley, 2013). These structural challenges lead to leadership avoidance as a strategic act of resource preservation and identity protection. In contrast, the UK’s academic environment, though more resource-rich, also features significant gendered challenges. Women face managerialised, audit-driven cultures that prioritize measurable outputs and masculine leadership norms, creating a dual burden of overwork and under-recognition.

Coping efforts like mentoring and pastoral care, essential to departmental functioning, remain largely invisible and unrewarded (Acker, 2012; Husu, 2022). Despite formal equality policies, informal biases penalise women for deviating from gendered leadership norms (Van den Brink and Benschop, 2018; Burkinshaw and White, 2017).

Table 2 shows comparison of the UK and Pakistan as two contrasting academic systems to justify why the same variables are worth examining cross-nationally. It shows that role-related stress, leader identity threat, coping mechanisms (and whether coping is institutionally recognised), and role-congruity climate exist in both contexts, but they differ sharply in intensity and institutional mediation such that the UK tends to feature high demands with partial buffering through formal workload, wellbeing, and equality infrastructures alongside more implicit bias, whereas Pakistan tends to feature more severe and structurally reinforced pressures with weak support, overt patriarchal norms, and heavier domestic expectations on women; taken together, the table indicates that a UK–Pakistan comparison enables a robust test of whether the theorised stress-to-strain and threat-to-avoidance pathways are universal or context-contingent, and whether institutional capacity and gender ideology act as key moderators that amplify or dampen women academics’ strain and leadership avoidance.

**Table 2:** Rationale of the study’s context.

Variable	UK context	Pakistan context	Comparative rationale
Role-related stress	UK academics experience high workloads, performance pressures, and administrative overload. However, institutionalised support systems (e.g., workload models, HR wellness initiatives) partially buffer stress effects. Women still report higher strain due to gendered service expectations (Tytherleigh et al., 2007; Kinman and Jones, 2008).	Pakistani female academics face acute structural and socio-cultural stressors, poor administrative support, gender bias, limited autonomy, and domestic demands. Institutional supports are weak or symbolic (Shah et al., 2014; Lodhi et al., 2023).	Both contexts reflect role overload and ambiguity but differ in intensity and institutional mediation. Cross-context analysis tests the universality of COR theory in gendered academic systems and examines whether institutional capacity moderates stress-to-strain pathways.
Leader identity threat	In the UK, identity threat arises subtly through exclusion from informal networks and gendered perceptions of leadership prototypes. Women fear evaluation bias and reputational cost (Howe-Walsh and Turnbull, 2016; Quickfall, 2025).	In Pakistan, identity threat is overt, rooted in patriarchal hierarchies and cultural expectations that leadership is male. Women often internalise these norms, leading to stereotype-confirmation anxiety (Naseem et al., 2023; Abbas et al., 2023).	Studying both illuminates how identity threats vary along the spectrum of implicit vs. explicit bias. This comparative angle tests whether leader identity threat is universally predictive of avoidance or context-dependent.
Coping-capital legibility	UK women deploy both individual and collective coping, peer support, mentoring, and flexible work arrangements. Yet institutional recognition of such coping remains inconsistent (Quickfall, 2025).	Pakistani women rely heavily on personal endurance and self-sacrifice, with minimal institutional acknowledgment or structural accommodation (Saleem and Ajmal, 2018; Kirmani et al., 2024).	Both demonstrate gendered invisibility of coping work. Comparative focus tests whether institutional recognition (coping-capital legibility) moderates stress outcomes differently in high-resource vs. low-resource systems.
Role-congruity climate	UK academia displays subtle bias: leadership prototypes are masculine, but formal equality policies mitigate overt exclusion (O’Connor, 2014; Howe-Walsh and Turnbull, 2016).	Pakistani academia reflects entrenched patriarchal norms and explicit gender-role segregation in leadership roles (Bhatti and Ali, 2021; Kaka and Hashmi, 2025).	Joint analysis examines whether role-congruity pressures predict similar avoidance patterns across distinct gender ideologies. It tests Role-Congruity Theory’s cross-cultural validity in explaining women’s underrepresentation in leadership.

In this high-surveillance, low-legibility environment, leadership becomes a reputational risk rather than an opportunity, heightening identity threat and accelerating resource depletion cycles (Morley, 2013; Gill, 2009). Both contexts converge in producing gendered depletion spirals, where leadership avoidance emerges as an adaptive self-preservation strategy, albeit driven by distinct institutional logics. Pakistan’s overt patriarchy and rigid hierarchies contrast with the UK’s performative meritocracy, where gendered biases are embedded in institutional practices. For global higher education systems, understanding how institutional support (coping-capital legibility) and role-congruity climates interact with identity threat across socio-economic contexts informs targeted interventions. In Pakistan, this could drive capacity-building and gender policy reform, while in the UK, it could refine leadership development and mentoring programs (Acker, 2012).

### 2.4 Role stress, personal strain, and leader identity threat

To clarify the conceptual model, it is important to distinguish role stress, personal strain, and leader identity threat, because these constructs capture related but analytically distinct stages of the process leading to leadership avoidance. Role stress refers to the demands embedded in the academic role, such as overload, ambiguity, conflict, and responsibility pressures (Doyle and Hind, 2002; Tytherleigh et al., 2007). Personal strain refers to the depletion response that follows prolonged exposure to those demands, including fatigue, emotional exhaustion, and reduced coping capacity, consistent with Conservation of Resources theory (Hobfoll, 1989, 2018). Leader identity threat, by contrast, refers to the socially evaluative risk that one’s legitimacy, belonging, or acceptability as a leader may be questioned in gendered institutional environments where leadership remains associated with masculine prototypes (Eagly and Karau, 2002; Hoyt and Murphy, 2016). Distinguishing these constructs allows the model to specify more precisely how demanding role conditions may translate into leadership avoidance, whether directly, indirectly through depletion or identity risk, or sequentially through both.

The starting point of the model is the direct burden of role stress itself. Role stress refers to the demands embedded in the academic role, including overload, ambiguity, conflict, responsibility burden, and environmental pressures. In university settings, women academics often encounter these demands simultaneously because they must manage teaching, research, administration, and service obligations while also carrying gendered expectations around mentoring, caregiving, and institutional citizenship (Guarino and Borden, 2017; Williams et al., 2023). Role Theory suggests that when work roles become characterised by incompatible expectations, excessive demands, or unclear boundaries, individuals are more likely to disengage from additional responsibilities that would intensify those pressures (Doyle and Hind, 2002; Tytherleigh et al., 2007). Seen in this light, leadership avoidance can arise not only as an outcome of deeper internal processes, but also as a direct response to already burdensome role conditions.

High role stress is expected to be associated directly with greater leadership avoidance among women academics. This expectation follows from both Role Theory and Conservation of Resources (COR) theory. Role Theory suggests that when individuals face excessive, conflicting, or ambiguous demands, withdrawal becomes a rational response to unsustainable role pressure (Kahn et al., 1964; Rizzo et al., 1970). COR theory likewise proposes that individuals seek to preserve remaining resources when demands threaten to outstrip available energy, time, and emotional capacity (Hobfoll, 1989, 2018). In university settings, where women frequently carry disproportionate service, pastoral, and relational obligations in addition to core teaching and research work, leadership may reasonably be judged as an additional source of workload, exposure, and responsibility rather than as an attractive opportunity (Gill, 2009; Guarino and Borden, 2017; O’Connor, 2014; Williams et al., 2023). Under such conditions, avoiding leadership is not best interpreted as a lack of ambition, but as a protective response to already burdensome role conditions.

*Hypothesis 1a*: Higher role stress is positively associated with leadership avoidance.

A second step in the argument requires differentiating role stress from personal strain. Whereas role stress captures the demands or pressures associated with one’s work role, personal strain refers to the psychological and physiological depletion that follows prolonged exposure to those demands. It is reflected in fatigue, irritability, reduced coping capacity, emotional exhaustion, and spillover into broader functioning. In other words, strain is not the demand itself but the depletion outcome produced when role demands consume time, energy, and emotional resources over time. This distinction is central to COR theory, which argues that continued exposure to demanding conditions generates resource loss and depletion rather than remaining external to the individual (Hobfoll, 1989, 2018). Existing work in academic settings similarly shows that chronic job demands are associated with burnout, fatigue, lower job satisfaction, and reduced motivation, especially where women carry disproportionate relational and administrative labour that is weakly rewarded (Fauzi et al., 2024; Kinman and Jones, 2008; Yahya et al., 2024).

Role stress is also expected to increase personal strain. Conceptually, this is the first step in the proposed indirect process. Role stress reflects the external pressures embedded in the work role, whereas personal strain reflects the depletion response that follows prolonged exposure to those pressures. COR theory is particularly useful here because it explains why persistent demands consume valued resources and generate fatigue, emotional exhaustion, and reduced coping capacity over time (Hobfoll, 1989, 2018). Role Theory similarly suggests that overload, conflict, and ambiguity do not remain external to the individual, but are experienced psychologically when expectations become difficult to reconcile or sustain (Tytherleigh et al., 2007). For women academics, this process may be especially pronounced because formal role demands often coexist with less visible but highly consequential relational and institutional expectations, including mentoring, student support, and organisational citizenship work that is not always adequately recognised (Fauzi et al., 2024; Kinman and Jones, 2008; Yahya et al., 2024). This makes it theoretically reasonable to expect that higher role stress will be accompanied by greater personal strain, and that such strain will in turn make leadership avoidance more likely because depleted individuals are less able and less willing to absorb the additional costs associated with formal leadership roles.

*Hypothesis 1b:* Higher role stress is positively associated with personal strain, which in turn is positively associated with leadership avoidance. Accordingly, role stress has a positive indirect effect on leadership avoidance via personal strain.

The model also requires distinguishing personal strain from leader identity threat. Strain captures a depleted psychological state, whereas leader identity threat captures a socially evaluative concern that one’s legitimacy, belonging, or acceptability may be undermined in the leadership domain. For women, this threat arises because leadership continues to be associated with masculine prototypes, creating tension between gendered expectations and leader role expectations (Eagly and Karau, 2002; Hoyt and Murphy, 2016). In academic institutions, where leadership is often masculinised, women may anticipate negative judgement, exclusion, or backlash if they enact authority or signal leadership ambition (Acker, 2012; Carnes et al., 2015; O’Connor, 2014). A woman academic may therefore feel strained because of workload without necessarily experiencing identity threat, but identity threat becomes more salient when leadership itself is seen as socially risky and normatively incongruent.

Role stress is also expected to heighten leader identity threat, which provides a second indirect pathway to leadership avoidance. Whereas the strain pathway captures depletion, this pathway captures the socially evaluative and identity-relevant risks attached to leadership in gendered institutional environments. Role-Congruity Theory argues that leadership remains normatively associated with masculine traits, creating a mismatch between expectations of femininity and expectations of leadership (Eagly and Karau, 2002). In academic institutions, where leadership is often masculinised, women may anticipate negative judgement, exclusion, or backlash when they display authority or signal leadership ambition (Acker, 2012; Carnes et al., 2015; O’Connor, 2014). Higher role stress may intensify this process because increasing workload, visibility, and responsibility can make the prospect of leadership feel not only more demanding, but also more exposed to social evaluation. Recent scholarship likewise indicates that gendered evaluative climates in universities sustain identity-relevant risk appraisals for women who contemplate leadership participation (Meza-Mejia et al., 2023; Quickfall, 2025). It is therefore theoretically plausible that role stress increases leader identity threat and that this heightened threat contributes to stronger leadership avoidance.

*Hypothesis 1c:* Higher role stress is positively associated with leader identity threat, which in turn is positively associated with leadership avoidance. Accordingly, role stress has a positive indirect effect on leadership avoidance via leader identity threat.

Bringing these constructs together, the model proposes a sequential rather than an additive process. First, role stress reflects the highly demanding conditions of academic work. Second, sustained exposure to those demands depletes valued resources and produces personal strain. Third, once individuals are strained and have fewer psychological and emotional reserves available, the prospect of entering leadership becomes more identity-relevant and more threatening because leadership in gendered environments entails heightened visibility, scrutiny, and possible backlash. COR theory supports this logic by suggesting that depleted individuals become more protective of their remaining resources and more sensitive to contexts that may trigger additional loss (Hobfoll, 1989, 2018). Role-Congruity Theory complements this argument by suggesting that women are especially likely to perceive leadership as identity-risking when leadership norms are incongruent with femininity (Eagly and Karau, 2002). The sequential claim is therefore that strain and identity threat are distinct but connected stages in the transmission of role stress into leadership avoidance.

The model proposes that these two mechanisms can operate sequentially rather than independently. The theoretical logic is that sustained role stress first depletes personal resources, producing strain, and that this depleted condition then heightens sensitivity to leader identity threat. Put differently, women academics who are already strained may become more vulnerable to the social and psychological risks attached to leadership because they have fewer emotional and cognitive resources with which to absorb scrutiny, contestation, and possible backlash. COR theory supports this ordering by suggesting that depleted individuals become more protective of remaining resources and more alert to contexts that may trigger further loss (Hobfoll, 1989, 2018). Role-Congruity Theory complements this argument by explaining why leadership is especially likely to be experienced as identity-risking for women in masculinised institutional settings (Eagly and Karau, 2002). The sequential argument therefore is not that strain and identity threat are interchangeable, but that depletion can intensify vulnerability to identity-relevant concerns, which in turn makes leadership avoidance more likely. This sequential pathway is particularly relevant in higher education, where women often confront both chronic workload pressure and gendered evaluative climates at the same time (Avolio et al., 2024; Harris et al., 2024; Tang and Horta, 2024).

*H1d (sequential indirect effect):* Higher role stress is positively associated with personal strain, which is positively associated with leader identity threat, which in turn is positively associated with leadership avoidance. Accordingly, role stress has a positive sequential indirect effect on leadership avoidance via personal strain and leader identity threat.

### 2.5 Coping mechanisms and coping-capital legibility

COR theory posits that individuals use coping to prevent further resource loss, but organisational recognition of coping determines whether these strategies replenish or further deplete resources. The concept of coping-capital legibility (Husu, 2022; Kelly et al., 2011) extends this by emphasising how institutions reward or ignore these invisible forms of work.

In this study, legibility is used in its institutional sense: the extent to which a practice is rendered visible, noticed, classifiable, and “countable” within an organisation’s formal setting. Classic accounts of legibility emphasise that powerful institutions allocate attention and resources to what they can systematically “see” through categories, metrics, and records, referred to as visibility by Yahya et al. (2024) between male and female instructors. In universities, these legibility devices include workload allocation models, promotion rubrics, performance indicators, and committee reporting. When coping labour is legible (visible), it becomes eligible for recognition, valuation, and redistribution.

This is why we treat institutional recognition of efforts spent in coping as a theoretically appropriate, proximal indicator of coping-capital legibility rather than a competing construct. Recognition is the observable consequence of legibility: practices that are made legible can be credited, legitimised, and exchanged for valued returns (for example workload relief, sponsorship, promotion credit, or reputational standing). Conversely, where coping labour is illegible, it is more likely to be treated as naturalised “women’s service,” producing resource outflow without organisational return, consistent with research on invisible labour and gendered organisational processes (Acker, 2012; Husu, 2022). In short, legibility is the organisational precondition, and leads onto recognition and valuation as downstream outcomes. Because our aim is to capture women’s perceived exposure to that recognition system, our items deliberately target whether mentoring, relational co-ordination, and emotional labour are visible and credited in the institutional accounting architecture.

Acker (2012), for example, highlights how caring and relational labour within universities is frequently normalised as part of women’s roles rather than formally recognised as a contribution to institutional performance. Similarly, Babcock et al. (2017) demonstrate that women are more frequently asked to undertake low promotability tasks that sustain organisational operations but are rarely rewarded in formal advancement structures. When these forms of labour remain outside institutional accounting systems, they generate effort without producing career capital. Women academics often engage in mentoring, relational coordination, and emotional support as strategies to manage workplace pressures, identity strain, and structural barriers in academic environments characterised by high workload and gendered expectations, as noted by Barkhuizen and Rothmann (2008) in studies of academic occupational stress.

Mentoring can function as coping when it is used to manage stress exposure and identity strain, not when it is used as a formal leadership exercise. For women academics, mentoring can operate as a resource-protective strategy in at least four ways. First, it builds reciprocal support ties that provide emotional validation, practical problem-solving, and vicarious learning, which buffer strain under chronic workload and evaluative pressure. Second, mentoring relationships can create informal sponsorship and information channels that reduce uncertainty and reputational risk in masculinised leadership climates, thereby lowering identity threat. Third, mentoring junior women can serve as identity affirmation, restoring a sense of competence and belonging when leadership prototypes are gender-incongruent. Fourth, collective mentoring circles distribute emotional labour and reduce isolation, which is a known amplifier of loss of resources, spirals in audit-heavy academic settings (Harris et al., 2024; Tang and Horta, 2024). However, the same coping behaviour becomes resource-draining when institutions treat mentoring as “natural female service” rather than creditable work, because time and emotional effort are expended without workload relief or advancement value (Fauzi et al., 2024; Yahya et al., 2024). This is precisely why the model distinguishes coping behaviour from coping-capital legibility, which captures whether coping is institutionally convertible into replenishing capital.

Coping-capital legibility shares similarities with emotional labor in that both focus on the invisibility of essential labor, such as mentoring and emotional support, often expected of women in academic environments (Acker, 2012; Hochschild, 1983). Both concepts highlight the gendered nature of these tasks, where women are disproportionately expected to perform labor that sustains institutional functioning, but without institutional recognition or reward. Like emotional labor, when these efforts are invisible, they can lead to resource loss and burnout, especially for women in academia (Hochschild, 1983; Husu, 2022).

However, coping-capital legibility is distinct in its focus on how coping behaviors such as emotional regulation or service work are institutionally recognised and rewarded (Acker, 2012; Kelly et al., 2011). Unlike emotional labor, which primarily deals with emotional regulation to meet organisational demands, coping-capital legibility specifically examines whether these efforts convert into tangible benefits like sponsorship, workload relief, or career advancement, which are critical for sustaining leadership aspirations. This conversion into capital makes coping-capital legibility a broader concept that links individual coping efforts to institutional evaluation systems, a dimension not typically addressed in emotional labor theory.

Quickfall (2025) highlighted how women in English universities engaged in extensive emotional and mentoring labour, yet these were undervalued in promotion systems. Doyle and Hind (2002) and Van Emmerik (2002) found that social support mitigated burnout more effectively for women, illustrating that recognised coping behaviours strengthen resilience.

Saleem and Ajmal (2018) reported that Pakistani women cope through personal effort and overextension rather than institutional recourse, reinforcing the invisibility of their contributions. Lodhi et al. (2023) and Kirmani et al. (2024) further linked unacknowledged coping to exhaustion and reduced performance. Despite resilience, the absence of formal recognition limits coping’s restorative function. In the UK, institutional recognition remains partial but present, whereas in Pakistan it is virtually absent. This divergence reflects different levels of coping-capital legibility. When coping is recognised, women sustain performance with less strain; when it is ignored, coping accelerates burnout and avoidance. Thus, coping-capital legibility operates as a contextual moderator between role stress and strain.

*Hypothesis 2a:* Coping-capital legibility moderates the role stress to personal strain path such that the positive association is weaker when coping-capital legibility is high and stronger when coping-capital legibility is low.

*H2b (conditional sequential indirect):* The sequential indirect effect of role stress on leadership avoidance via personal strain and leader identity threat is stronger when coping-capital legibility is low and weaker when coping-capital legibility is high.

### 2.6 Role-congruity climate

Role-Congruity Theory (Eagly and Karau, 2002) explains that prejudice arises when leadership roles are seen as incongruent with female gender roles. In academic contexts, masculinised leadership ideals penalise women for both displaying and refraining from assertive behaviours. This incongruity alters cost–benefit appraisals, making leadership pursuit riskier.

O’Connor (2014) and Howe-Walsh and Turnbull (2016) show that British academic institutions remain gendered in their leadership norms. Women often downplay ambition to avoid backlash. Quickfall (2025) confirms that women in English universities perceive leadership prototypes as masculine, which fosters disengagement.

In Pakistan, gendered role-congruity pressures are entrenched. Bhatti and Ali (2021) found that female academic heads were compelled to emulate male traits to gain legitimacy. Farooq et al. (2020) revealed that exclusionary cultures and lack of role models reinforce this incongruity. Kaka and Hashmi (2025) reported that gender-biased climates deter leadership pursuit, maintaining male dominance.

**Figure 2:**
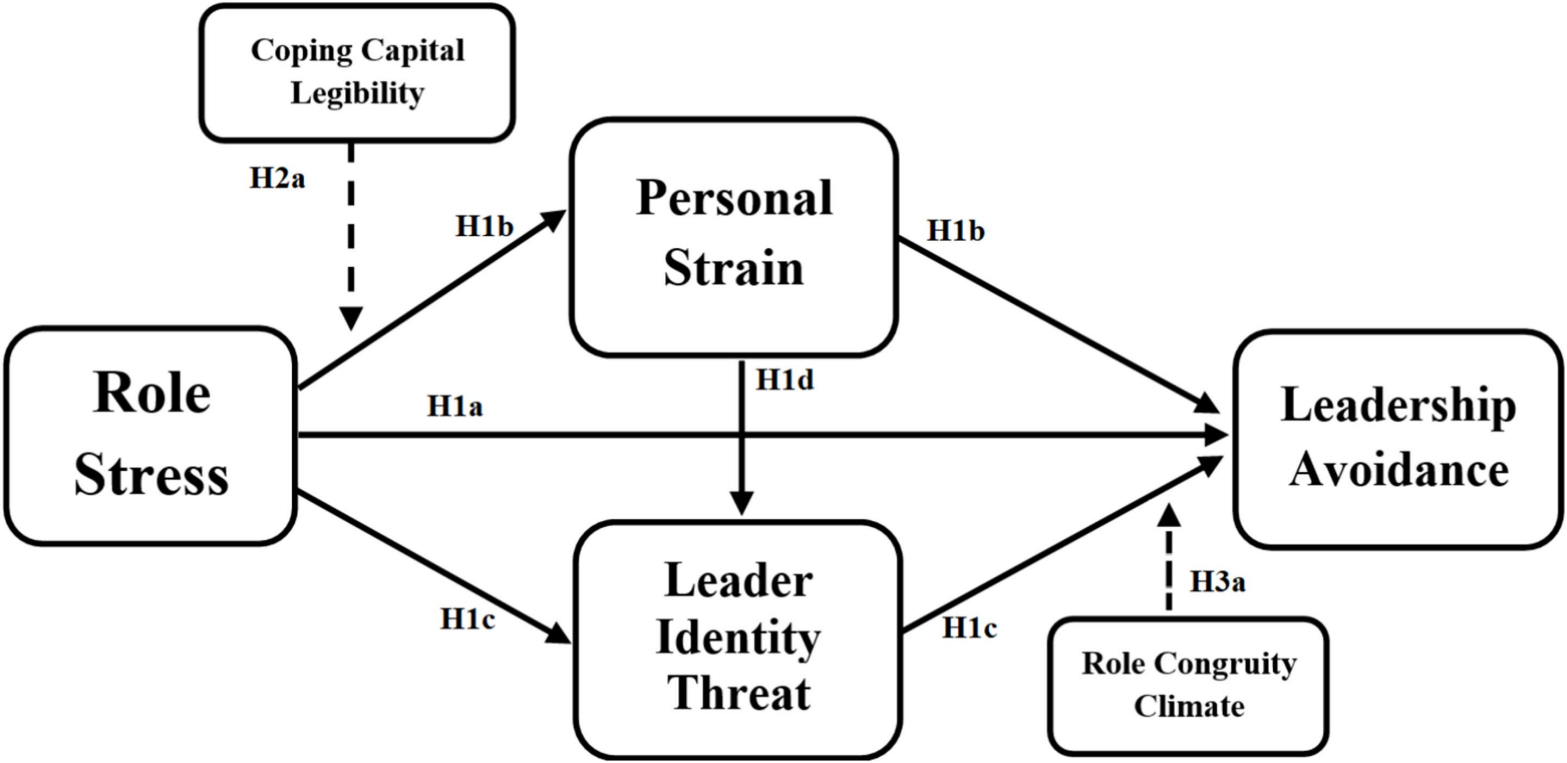
Conceptual model of this study.

Across both systems, women’s leadership trajectories are constrained by masculinised norms, though the severity is context-specific. British academia reflects institutionalised subtle bias, whilst Pakistan’s reflects structural patriarchy. In both, incongruity between gender identity and leadership expectations elevates identity threat and avoidance (see Figure 2).

*Hypothesis 3a:* Role-congruity climate moderates the leader identity threat to leadership avoidance path such that the positive association is stronger when role-congruity climate is high and weaker when role-congruity climate is low.

*Hypothesis 3b:* The sequential indirect effect of role stress on leadership avoidance via personal strain and leader identity threat is stronger when role-congruity climate is high and weaker when role-congruity climate is low.

## 3 Methodology

### 3.1 Design, participants, sample and procedures

We employed a quantitative, cross-sectional, comparative design to examine how occupational stress and institutional conditions jointly shape women academics’ leadership avoidance in two contrasting higher education systems: the United Kingdom (UK) and Pakistan. The model integrates the Occupational Stress Inventory-Revised (OSI-R; Osipow, 1998) architecture as a scaffold for operationalising *r*ole stress and resultant personal strain due to loss of mental resources, augmented with constructs capturing leader identity threat, coping-capital legibility, role-congruity climate, and leadership avoidance. The study focused exclusively on women faculty, as the theorised mechanism, stress-induced leadership avoidance, reflects gendered occupational and identity dynamics. Comparative analyses across the two contexts test whether similar psychological processes manifest differently under distinct institutional and cultural logics.

We used a cross-sectional design because the study’s objective is prediction testing under clearly specified theoretical ordering. The study sought to capture women academics’ perceptions of role stress, personal strain, leader identity threat, coping-capital legibility, role-congruity climate, and leadership avoidance within the same institutional period across two national higher education systems. A cross-sectional survey was appropriate for this purpose because it enabled comparable data collection from matched groups in the United Kingdom and Pakistan and allowed us to examine whether the theorised pattern of relationships was observable across contrasting contexts. We recognise that longitudinal or multiwave designs are better suited to establishing temporal sequencing and stronger causal claims. Accordingly, the present design is used to examine theory-informed associations among the focal constructs rather than to make definitive claims about temporal causality. The ordering of the variables is grounded in prior theory and literature, and the findings are interpreted with appropriate caution.

Accordingly, we treat mediation inference as theory-consistent rather than time-confirmed, and we report sensitivity checks against plausible alternative orderings in the Results robustness section.

Across the two countries, 847 potential respondents were invited (UK: 537; Pakistan: 310). The realised samples were UK *n* = 236 and Pakistan *n* = 229, yielding response rates of 43.94% (UK) and 73.87% (Pakistan), respectively. While our UK sample rate of 43.9% might appear modest compared to traditional survey benchmarks, response rate alone is not a definitive indicator of nonresponse bias in survey research (Narayanan et al., 2024; Groves and Peytcheva, 2008). Instead, the pattern of nonresponse and its relation to substantive variables determine bias risk, which is why we conducted robustness checks comparing early versus late responders on key constructs.

Specifically, we compared early versus late respondents on the core study constructs and key demographics (wave analysis) and found no practically meaningful differences. In addition, results were robust to alternative estimation specifications using cluster-robust standard errors at the university level and to the exclusion of influential cases. These checks reduce (but cannot eliminate) concerns that the observed effects reflect systematic non-response rather than the theorised relationships. The achieved sample sizes exceed commonly cited minimum thresholds for stable SEM estimation and multi-group comparisons (for example, group sizes above 200 for models of moderate complexity). Seminal methodological authorities support a conventional benchmark of *N* > 200 as adequate for structural equation modelling in behavioural research (Kline, 2016).

Data for the Pakistan cohort were drawn from 22 public-sector universities across five major cities (Islamabad, Rawalpindi, Lahore, Karachi, and Peshawar), representing all four provinces. Using stratified random sampling, we ensured coverage across rank, discipline, and university type. Four trained female research assistants conducted data collection between June 2024 and March 2025, distributing both paper-based and online (Qualtrics) surveys with prior administrative approval from university HR offices. The realised Pakistani sample included 229 women academics. Ages ranged from 26 to 61 years (*M* = 42.5, SD = 8.1). Academic rank distribution was Lecturer (41.1%), Assistant Professor (36.0%), Associate Professor (15.3%), and Professor (7.6%). The disciplinary composition reflected national representation: Management and Social Sciences (23.3%), Humanities (18.2%), Psychology and Education (16.5%), Hard Sciences (21.4%), and STEM (20.6%). Department identifiers were recorded to enable potential climate aggregation analyses (Guarino and Borden, 2017; Williams et al., 2023).

Parallel data were collected from 19 UK universities, including both research-intensive (Russell Group) and post-1992 institutions, to capture structural variation in academic climates. Recruitment was conducted through professional mailing lists, institutional staff networks, and social media groups for women in academia (e.g., Advance HE, Aurora Programme). The UK sample comprised 236 women faculty. Ages ranged from 28 to 63 years (*M* = 44.2, SD = 7.7). Rank composition was Lecturer (33.5%), Senior Lecturer (29.2%), Reader/Associate Professor (25.8%), and Professor (11.5%). Academic disciplines included Education (20.8%), Management and Social Sciences (24.5%), Humanities (18.9%), and STEM (35.8%). Data were collected between August 2024 and June 2025, entirely via online surveys.

Language of administration and pretest procedure: All measures were administered in English in both the UK and Pakistan samples, consistent with the professional working language of university faculty (teaching, administrative documentation, and research communication). In Pakistan, English is the working language, all books/syllabi are in English and medium of instruction in universities is also English, and official correspondence carried out in English as well. To mitigate comprehension risk and ensure linguistic clarity, we conducted a pretest with women academics in both contexts prior to full launch, focusing on item readability, contextual appropriateness, and interpretive equivalence. Only minor wording adjustments were made to improve clarity without altering construct meaning.

### 3.2 Ethics and consent

Ethical clearance was obtained from the authors’ university ethics committees and the permissions were obtained from participating institutions. Participation was voluntary, anonymous, and conducted in accordance with the Declaration of Helsinki (2013). All participants received information sheets detailing the study purpose, confidentiality protections, and their right to withdraw without consequence. Completion of the survey implied informed consent. The research involved no deception, incentives, or invasive procedures, and participant risk was minimal.

### 3.3 Measures

All constructs were measured using multi-item instruments with five-point Likert-type response formats (1 = strongly disagree, 5 = strongly agree). Reliability evidence for each latent construct (Cronbach’s *α* and composite reliability) is reported in Table 3. All constructs were measured using five-point Likert-type scales (1 = strongly disagree, 5 = strongly agree). Measures were administered in English in both countries. This was appropriate because English is the medium of instruction and formal academic work in Pakistani higher education, and the instrument wording was pretested with academics in both samples to confirm clarity and conceptual equivalence.

**Table 3:** Reliability and convergent validity.

Construct	*α* (UK)	CR (UK)	*α* (PAK)	CR (PAK)	AVE (UK)	AVE (PAK)
Role stress	0.78	0.91	0.86	0.87	0.62	0.67
Personal strain	0.79	0.90	0.88	0.89	0.69	0.74
Leader identity threat	0.74	0.81	0.84	0.85	0.64	0.58
Coping-capital legibility	0.77	0.88	0.80	0.81	0.58	0.69
Role-congruity climate	0.79	0.84	0.73	0.86	0.68	0.60
Leadership avoidance	0.73	0.83	0.85	0.86	0.66	0.71

*α*, Cronbach’s Alpha, CR, composite reliability.

#### 3.3.1 Role stress

Measured using the Occupational Stress Inventory-Revised (OSI-R) Occupational Roles Questionnaire (Osipow, 1998). The scale captures chronic role-based demands including role overload, role ambiguity, inter-role conflict, responsibility, and environmental stressors. Sample item: “I have too many work responsibilities to handle.”

#### 3.3.2 Personal strain

Measured using the OSI-R Personal Strain Questionnaire (Osipow, 1998), covering psychological, interpersonal, vocational, and physical strain reactions to sustained demands. Sample item: “Recently, I feel emotionally drained by my work.” These OSI-R subdomains have previously been applied in educator samples, supporting their relevance for academic work contexts (Barkhuizen and Rothmann, 2008).

#### 3.3.3 Leader identity threat

Leader identity threat captured perceived risk of negative judgement, penalty, or exclusion when enacting agentic leadership behaviours in gendered contexts. The items were grounded in role congruity and stereotype threat logic (Eagly and Carli, 2007; Hoyt and Murphy, 2016). Sample item: “Acting assertively here risks negative evaluations.”

#### 3.3.4 Coping-capital legibility

Coping-capital legibility captures the perceived extent to which women’s coping labour (for example mentoring, relational co-ordination, and emotional labour) is institutionally legible, meaning visible, recordable, and creditable within formal evaluative systems. Legibility is not a rhetorical label in this study. It denotes an institutional mechanism through which particular forms of work become eligible for recognition and value allocation. Organisational research shows that activities become rewardable when they are made administratively “seen” through categories, metrics, and commensuration processes. In universities, such legibility devices include workload models, promotion criteria, and performance reviews. When coping labour is legible to these devices, it is more likely to translate into valued returns (for example workload relief, sponsorship, or advancement credit). When it is illegible, the same labour is more likely to remain invisible and uncompensated, intensifying resource loss and reinforcing gendered patterns of undervaluation (Acker, 2012; Husu, 2022).

Accordingly, our operationalisation focuses on perceived recognition because recognition is the most direct organisational manifestation of legibility in day-to-day academic systems. Items therefore assess whether coping labour is acknowledged and credited in concrete institutional decisions (for example workload allocation and promotion). A sample item is: “Mentoring and relational work are recognised in workload or promotion decisions.” Higher scores indicate that coping labour is visible and institutionally creditable, and therefore more convertible into valued organisational capital. Lower scores indicate that coping work remains institutionally illegible and, as a result, more likely to be resource-draining rather than resource-replenishing (Kelly et al., 2011; Husu, 2022).

#### 3.3.5 Role-congruity climate

Role-congruity climate assessed perceptions that leadership roles are masculinised and that women’s authority is scrutinised, contested, or penalised, reflecting the meso-level climate within which identity threat is more likely to translate into withdrawal. This construct was grounded in role congruity theory and gendered leadership climate work (Eagly and Karau, 2002; Van den Brink and Benschop, 2018). Sample item: “Women who lead assertively are judged more harshly here than men.”

#### 3.3.6 Leadership avoidance

Leadership avoidance captured reluctance to apply for, accept, or sustain formal leadership roles, including behavioural withdrawal and cognitive disengagement (O’Connor, 2014). Sample items: “I prefer not to take on formal leadership responsibilities” and “Leadership here is not worth the personal cost.”

#### 3.3.7 Marker variable

We used a marker variable of a 3-item behavioural-intention construct adapted from Venkatesh et al. (2003), Intention to Use Contactless Payment by replacing ‘the system’ with ‘Contactless Payment’. Sample item; “I intend to use contactless technologies for my personal purchases in the next 6 months”. It provides a small comparison factor that should not meaningfully overlap with main variables. In case it shared variance with the main constructs, that may indicate common method bias (Williams et al., 2010).

### 3.4 Data screening and preliminary analyses

All responses were screened for missing data, multivariate outliers, and distributional assumptions. Missingness was handled using Full Information Maximum Likelihood (FIML) under the Missing at Random (MAR) assumption. Normality diagnostics indicated acceptable skewness and kurtosis (|*z*| < 2.0). Robust Maximum Likelihood Estimation (MLR) was used to accommodate minor deviations from normality (Yuan and Bentler, 2000; Enders and Bandalos, 2001).

### 3.5 Common method variance (CMV) diagnostics and reporting

Because the study uses single-source self-report survey data, we conducted two complementary CMV diagnostics: a marker variable technique (MVT) and an unmeasured latent method factor (ULMF) approach. Using both is deliberate; MVT (Table 4) evaluates whether shared method variance is detectable via a theoretically unrelated measured construct (observed-method bias), whereas the ULMF (Table 5) approach tests whether an unmeasured common factor accounts for covariance among indicators beyond substantive constructs (unobserved shared-method bias). Using both strengthens inference by triangulating CMV risk from two different angles (Podsakoff et al., 2003; Richardson et al., 2009; Williams et al., 2010).

**Table 4:** Marker variable technique (MVT) model comparisons.

Model	Specification	*χ* ^2^	df	CFI	TLI	RMSEA	SRMR	Δ*χ*^2^	Δdf	*p* (Δ*χ*^2^)	Interpretation
A	Baseline measurement model (traits only)	1,268	604	0.95	0.938	0.052	0.045	–	–	–	Baseline fit acceptable
B	Marker factor added (free loadings)	1,246	596	0.953	0.942	0.05	0.043	22.2	8	0.004	Small fit improvement
C	Marker loadings constrained equal	1,259	603	0.951	0.939	0.051	0.044	12.7	7	0.078	No significant loss of fit vs. B
U	Unconstrained method model	1,239	592	0.955	0.944	0.049	0.042	29.9	12	0.003	Slight incremental improvement
R	Restricted method model	1,250	600	0.952	0.941	0.05	0.043	11.2	8	0.191	No meaningful decrement vs. U

*χ*^2^ = robust scaled chi-square (MLR estimator). Nested comparisons use Satorra–Bentler scaled *χ*^2^ difference tests (Williams et al., 2010).

**Table 5:** Unmeasured latent method factor (ULMF) results.

Model	*χ* ^2^	df	CFI	TLI	RMSEA	SRMR	Δ*χ*^2^	Δdf	*p* (Δ*χ*^2^)	Method variance (%)	Substantive paths stable?
Baseline CFA (traits only)	1268.4	604	0.95	0.94	0.052	0.045	–	–	–	–	–
ULMF model (method factor added)	1235.9	592	0.96	0.94	0.049	0.042	32.5	12	0.001	6.80%	Yes
ULMF + structural paths	1578.4	710	0.95	0.93	0.052	0.046	–	–	–	6.50%	No material change

Method factor specified orthogonal to substantive factors; method loadings constrained equal within construct for identification (Podsakoff et al., 2003; Richardson et al., 2009). Nested comparisons use robust Satorra–Bentler scaled *χ*^2^ difference testing.

MVT specification and required model set: The full CFA-marker model set (A, B, C, U, R) was estimated following Williams et al. (2010). Model A represents the baseline seven-factor measurement structure. Adding the marker factor with freely estimated loadings (Model B) resulted in a statistically significant but substantively modest improvement in model fit (Δ*χ*^2^ = 22.2, Δdf = 8, *p* = 0.004). Constraining marker loadings to equality (Model C) did not significantly worsen fit relative to Model B (Δ*χ*^2^ = 12.7, Δdf = 7, *p* = 0.078), suggesting that any method effects were relatively uniform across indicators. The unconstrained method model (Model U) showed marginal improvement over Model A; however, the restricted model (Model R) did not significantly differ from Model U (Δ*χ*^2^ = 11.2, Δdf = 8, *p* = 0.191), and substantive structural paths remained stable across specifications. The pattern indicates that although minor shared variance may be present, common method variance does not materially bias substantive relationships.

As a second diagnostic, we estimated a ULMF model by adding a latent method factor that loads on all observed indicators in the measurement model while retaining the substantive latent factors. To support model identification and interpretability, method factor loadings were constrained equal within construct and the method factor was specified as orthogonal to substantive factors (Podsakoff et al., 2003; Richardson et al., 2009). We report (a) comparative fit relative to the baseline CFA, (b) proportion of variance attributable to the method factor and (c) whether key substantive paths and indirect effects change in magnitude or significance under method control. If substantive estimates remain stable, this supports the conclusion that CMV is unlikely to be driving the central inferences.

## 4 Results

### 4.1 Descriptive patterns

Respondents from the UK reported higher *role stress* (*M* = 26.5, SD = 4.2) and *personal strain* (*M* = 25.5, SD = 4.0) than those from Pakistan (*M* = 24.5, SD = 3.9; *M* = 23.0, SD = 3.6, respectively). Parallel trends emerged for the extended constructs: UK respondents reported greater *leader identity threat* and *leadership avoidance*, alongside reduced *coping-capital legibility*.

### 4.2 Measurement model and invariance

A multi-group confirmatory factor analysis (CFA) evaluated the measurement model’s reliability and validity across both samples using MPlus 8.0 software. All latent constructs demonstrated satisfactory internal consistency. Convergent validity (Table 3) was evidenced by AVE > 0.50 for each factor (Table 3). A baseline CFA supported the seven-factor measurement model in the pooled sample, followed by multi-group CFA to evaluate cross-country equivalence. Fit indices for the baseline CFA, invariance steps, and the final structural model are reported in Table 6. Discriminant validity is reported in Table 7 per recommendations by Fornell and Larcker (1981). Configural invariance indicated the same factor structure across the UK and Pakistan samples. Metric and scalar constraints produced ΔCFI values below 0.01 and ΔRMSEA values below 0.005, supporting metric and scalar invariance and justifying cross-group comparisons of latent means and structural paths (Cheung and Rensvold, 2002; Hu and Bentler, 1999). These patterns suggest a resource-deficit and identity-threat profile among UK academics, aligning with loss-spiral processes in high-demand professional settings (Hobfoll, 1989; Barkhuizen and Rothmann, 2008) (see Table 8).

**Table 6:** Measurement invariance decision summary (UK vs. Pakistan).

Step	Constraints	CFI	RMSEA	SRMR	ΔCFI	ΔRMSEA	Decision
Configural	Same factor structure	0.948	0.051	0.044	–	–	Established
Metric	+ Equal loadings	0.939	0.053	0.048	0.009	0.002	Invariance held
Scalar	+ Equal intercepts	0.931	0.056	0.05	0.008	0.003	Invariance held

ΔCFI, change in comparative fit index; TLI, Tucker, Lewis index; RMSEA, root mean square error of approximation; SRMR, standardised root mean square residual.

**Table 7:** Discriminant validity.

Variables	RS	PS	LIT	CCL	RCC	LA	RS	PS	LIT	CCL	RCC	LA
	UK											
RS	*0.79*						*0.82*					
PS	0.66	*0.83*					0.58	*0.86*				
LIT	0.60	0.61	*0.80*				0.52	0.52	*0.76*			
CCL	−0.33	−0.35	−0.38	*0.76*			−0.27	−0.3	−0.32	*0.83*		
RCC	−0.28	0.42	0.20	0.38	*0.83*		−0.19	0.29	0.14	0.26	*0.78*	
LA	0.65	0.55	0.63	−0.36	0.19	*0.81*	0.55	0.48	0.52	−0.3	−0.13	*0.84*

RS, role stress; PS, personal strain; LIT, leader identity threat; CCL, coping-capital legibility; LA, leadership avoidance. Diagonal values are √AVE; off-diagonals are latent correlations.

**Table 8:** Model fit indices across CFA, invariance, and SEM models.

Model	Estimator	*χ* ^2^	df	*χ*^2^/df	CFI	TLI	RMSEA	RMSEA 90% CI	SRMR	AIC	BIC
A1. Baseline CFA (7-factor measurement model, pooled)	MLR	1268.4	604	2.1	0.95	0.938	0.052	[0.048, 0.056]	0.045	38984.2	39412.7
A2. Multi-group CFA: Configural invariance (UK vs. PAK)	MLR	1321.6	614	2.15	0.948	0.936	0.051	[0.047, 0.055]	0.044	39031.5	39504.1
A3. Multi-group CFA: metric invariance (loadings constrained)	MLR	1389.9	628	2.21	0.939	0.927	0.053	[0.049, 0.057]	0.048	39086.8	39,519
A4. Multi-group CFA: Scalar invariance (loadings + intercepts constrained)	MLR	1468.8	644	2.28	0.931	0.92	0.056	[0.052, 0.060]	0.05	39144.7	39540.6
A5. Multi-group structural SEM (moderated sequential mediation)	MLR	1586.2	722	2.2	0.944	0.932	0.053	[0.049, 0.057]	0.047	39231.1	39744.2
A6. Causal reversal check (identity threat—strain)	MLR	2058.7	722	2.85	0.92	0.91	0.065	[0.061, 0.069]	0.061	39690.5	40203.6
A7. Controls added (age, tenure, institutional type)	MLR	1508.5	718	2.1	0.951	0.941	0.05	[0.046, 0.054]	0.045	39190.7	39705.3

*χ*^2^/df (Chi-square/degrees of freedom); Estimator MLR implies robust (scaled) *χ*^2^ and robust fit indices. Rules of thumb: CFI/TLI ≳ 0.90 (ideally ≳ 0.95), RMSEA ≲ 0.06, SRMR ≲ 0.08.

### 4.3 Structural model assessment

The hypothesised model was tested using multi-group structural equation modelling (SEM) with bootstrapped confidence intervals (5,000 resamples). Core structural paths were statistically significant in both samples. Role stress showed a strong positive association with leadership avoidance and also predicted personal strain and leader identity threat. Personal Strain predicted both identity threat and avoidance. Identity threat was positively associated with leadership avoidance in both contexts. Between-group *z*-diff values indicated that these core paths were consistently stronger in the UK than Pakistan (*z*-diff range: 1.97–2.32). Direct effects were evaluated using bootstrapped confidence intervals (Table 9). Both specific indirect pathways were significant in both samples: stress—strain—avoidance and stress—threat—avoidance.

**Table 9:** Path estimates and moderators (between-group test).

Path	UK (*n* = 236)	PAK (*n* = 229)	*z*-diff
Role stress—leadership avoidance	0.52*** (0.08)	0.43*** (0.07)	2.32
Personal strain—avoidance	0.35*** (0.07)	0.28*** (0.08)	1.97
Role stress—identity threat	0.40*** (0.08)	0.32*** (0.09)	2.09
Role stress—personal strain	0.54*** (0.07)	0.41*** (0.08)	1.98
Personal strain—identity threat	0.49*** (0.09)	0.32** (0.10)	1.99
Identity threat—leadership avoidance	0.46*** (0.08)	0.28* (0.11)	1.98
Stress × coping-capital legibility—strain	−0.096 (0.06)	−0.12 (0.08)	0.54
Threat × role-congruity climate—avoidance	0.22* (0.09)	0.15 (0.10)	0.53

*p < 0.05, **p < 0.01, ***p < 0.001.

For the sequential indirect pathway (stress—strain—threat—avoidance), the effect was clearly supported in the UK, but not supported in Pakistan. Conditional indirect effects are reported in Table 10. The interaction between stress and coping-capital legibility predicting Strain was negative in both samples but did not meet conventional significance, and between-group differences exist. Despite the weak interaction evidence, the conditional sequential indirect estimates show a consistent attenuation pattern: under low legibility, the sequential indirect was stronger but under high legibility it was reduced and became non-significant in Pakistan.

**Table 10:** Results for conditional and indirect effects.

Effect/path	Country/condition	Est. (*β*)	95% CI (LL–UL)	Sig.
H1a role stress—leadership avoidance	UK	0.52	[0.39, 0.65]	*p* < 0.01
	PAK	0.43	[0.32, 0.54]	*p* < 0.05
H1b specific indirect: stress—strain—leadership avoidance	UK	0.189**	[0.09, 0.27]	*p* < 0.01
	PAK	0.115*	[0.02, 0.23]	*p* < 0.05
H1c specific indirect: stress—threat—leadership avoidance	UK	0.184**	[0.07, 0.28]	*p* < 0.01
	PAK	0.094*	[0.02, 0.20]	*p* < 0.05
H1d sequential indirect: stress—strain—threat—leadership avoidance	UK	0.122	[0.054, 0.190]	*p* < 0.001
	PAK	0.037	[−0.002, 0.076]	ns
H2 conditional sequential indirect (legibility moderates stress—strain)	Low legibility (UK, *M* = −2)	0.165**	[0.07, 0.27]	*p* < 0.01
	High legibility (UK, *M* = +2)	0.078*	[0.01, 0.16]	*p* < 0.05
	Low legibility (PAK, *M* = −2)	0.058*	[0.01, 0.13]	*p* < 0.05
	High legibility (PAK, *M* = +2)	0.015	[−0.003, 0.053]	ns
H3 conditional sequential indirect (role-congruity moderates threat—avoidance)	Low role-congruity (UK, *M* = −1)	0.06	[−0.01, 0.14]	ns
	High role-congruity (UK, *M* = +1)	0.18**	[0.06, 0.26]	*p* < 0.01
	Low role-congruity (PAK, *M* = −1)	0.017	[−0.03, 0.09]	ns
	High Role-Congruity (PAK, *M* = +1)	0.056	[0.01, 0.12]	*p* < 0.05
UK Total indirect (sum of distinct indirects: H1b + H1c + H1 sequential)		0.495	[0.348, 0.641]	*p* < 0.001
PAK Total indirect (sum of distinct indirects: H1b + H1c + H1 sequential)		0.241	[0.120, 0.363]	*p* < 0.001
UK Total effect (direct + total indirect)		1.015	[0.800, 1.229]	*p* < 0.001
PAK Total effect (direct + total indirect)		0.671	[0.488, 0.854]	*p* < 0.001

*p < 0.05, **p < 0.01.

Under low role-congruity, the conditional sequential indirect is not reliably different from zero in the UK and is negligible and non-significant in Pakistan; under high role-congruity, the conditional sequential indirect is stronger in the UK and modest but significant in Pakistan. Overall, the mediated influence of stress on avoidance is substantial and statistically significant in the UK and is significant but smaller in Pakistan, and the combined direct plus mediated impact of stress on avoidance is very strong in the UK and strong and significant in Pakistan.

### 4.4 Latent mean comparisons

Scalar-invariant latent-mean tests revealed systematic national differences. UK participants scored higher on role stress, personal strain, identity threat, and leadership avoidance, whilst scoring lower on coping-capital legibility. The composite profile portrays UK academics as facing more acute role overload and identity tension, consistent with intensified institutional demands and performativity pressures in UK higher education (Morley, 2013). These differences correspond with the stronger indirect and conditional effects observed in the UK structural paths, suggesting that contextual and cultural factors amplify stress propagation and avoidance tendencies (see Table 11).

**Table 11:** Scalar-invariant latent-mean tests.

Construct	Δ*M* (UK-PAK)	S.E.	z
Role stress	0.420***	0.111	3.78
Personal strain	0.380***	0.11	3.46
Leader identity threat	0.360**	0.112	3.22
Coping-capital legibility	−0.270*	0.11	−2.450
Leadership avoidance	0.410***	0.113	3.64

Δ*M* values represent latent mean differences (UK minus Pakistan) under scalar invariance. S.E. values are reported to three decimal places. *p < 0.05, **p < 0.01, ***p < 0.001.

### 4.5 Robustness and sensitivity analyses

Model fit was examined through alternative specifications and diagnostic checks. Reversing the causal sequence (identity threat—strain) produced inferior model fit and smaller indirects. Common-method variance was tested using both a marker-variable and an unmeasured latent method-factor approach (Marsh et al., 2004); neither materially altered parameter estimates (Podsakoff et al., 2003). Furthermore, indirect effects remained stable after controlling for age, tenure, and institutional type, confirming the robustness of the structural patterns. Results affirm that UK respondents exhibit higher psychological strain and leadership avoidance, driven by greater role stress and leader-identity threat (see Table 12).

**Table 12:** Robustness and sensitivity analyses.

Test	(*χ*²/df)	Δ*χ*²/df	Fit indices
Causal reversal (identity threat—strain)	2.85	0.15	CFI = 0.92, TLI = 0.91, RMSEA = 0.065
Age, tenure, and institutional type control	2.10	–	CFI = 0.95, TLI = 0.94, RMSEA = 0.050

## 5 Discussion

The results show that leadership avoidance is not produced by a single factor but by a layered process in which demands, depletion, identity risk, and contextual conditions interact differently across the UK and Pakistan.

*Hypothesis 1a* predicted a positive association between role stress and leadership avoidance. This hypothesis was supported in both countries, with a stronger effect in the UK. This finding aligns with role theory and COR theory, both of which suggest that overload, ambiguity, and conflict make withdrawal more likely when demands exceed available resources (Hobfoll, 1989, 2018; Kahn et al., 1964; Rizzo et al., 1970). This may have emerged because women academics often experience leadership not as an attractive career step, but as an additional layer of responsibility on top of already demanding roles. It is also consistent with studies showing that women academics carry disproportionate service, mentoring, and boundary-spanning responsibilities, which can make leadership appear high-cost and low-return (Guarino and Borden, 2017; Williams et al., 2023). The finding therefore supports work arguing that women’s reluctance to pursue academic leadership is often a rational response to structural costs rather than a reflection of low aspiration (O’Connor, 2014). At the same time, the result should not be read deterministically. Wilkinson and Male (2025) found that senior women leaders in UK higher education responded to acute pressure during COVID through adaptive and distributed leadership rather than retreat, while Redmond et al. (2017) likewise showed that women could sustain and progress into leadership despite significant barriers. This suggests that role stress increases the likelihood of avoidance, but does not make it inevitable.

*Hypothesis 1b* proposed that role stress would predict leadership avoidance indirectly through personal strain. This hypothesis was supported in both samples. The result is consistent with COR theory, which treats strain as the depletion response to persistent demands, and with academic stress research linking chronic workload to burnout, fatigue, and lower motivation (Hobfoll, 1989, 2018; Kinman and Jones, 2008). This may have emerged because sustained role pressure can reduce the emotional and cognitive capacity needed to see leadership as manageable or worthwhile. It also fits evidence that women’s unpaid or weakly rewarded labour intensifies exhaustion in higher education settings (Fauzi et al., 2024; Yahya et al., 2024). However, the result also suggests that strain is one important pathway rather than the only pathway through which stress translates into avoidance. Harvey and Jones (2022) suggest that women’s leadership progression may be blocked not only by strain, but also by the weak documentation and recognition of distributed leadership work. Selzer and Robles (2019) similarly indicate that strategic self-positioning and leadership identity work may offset strain effects.

*Hypothesis 1c* predicted that role stress would also predict leadership avoidance indirectly through leader identity threat. This was supported in both countries. The result is in line with Role-Congruity Theory, which holds that women face greater evaluative risk when leadership is coded as masculine (Eagly and Karau, 2002). This may have emerged because role stress increases not only workload pressure, but also the perceived social risk of becoming visible in leadership. It also matches evidence that women in academic leadership settings face reputational penalties, exclusion, and scepticism when they signal authority or ambition (Carnes et al., 2015; Meza-Mejia et al., 2023). This helps explain why role stress is not merely a workload issue. It can also heighten the perceived identity cost of leadership. Still, the literature also offers a more complex picture. Gallant (2014) suggests that the issue may sometimes be less about identity threat itself and more about misfit between women’s self-positioning and institutional leadership scripts. Similarly, Wilkinson and Male (2025) show that women under pressure may continue leading by adapting their leadership style rather than avoiding leadership.

*Hypothesis 1d* predicted a sequential indirect effect from role stress to personal strain to leader identity threat to leadership avoidance. This hypothesis was clearly supported in the UK but not in Pakistan. The UK result supports the theorised ordering of the model: demands generate depletion, depletion heightens identity risk, and the combination encourages avoidance. This may have emerged because UK universities are shaped by managerial, audit-driven, and performance-intensive conditions in which workload pressure and subtle gender bias reinforce one another (Gill, 2009; Morley, 2013; Quickfall, 2025). The Pakistan result was weaker, because the full sequential effect was not significant even though the specific indirect effects through strain and threat were significant. A plausible explanation is that in Pakistan some barriers are sufficiently overt that women may conclude earlier that leadership is too costly, without needing the full internal sequence from strain to identity threat to unfold (Kirmani et al., 2024; Lodhi et al., 2023; Saleem and Ajmal, 2018). This does not invalidate the model, but suggests a more partial or compressed process. It also challenges the common assumption that more explicit patriarchy must necessarily produce the strongest complete pathway.

*Hypothesis 2a* proposed that coping-capital legibility would weaken the positive relationship between role stress and personal strain. The direct interaction term was directionally consistent in both countries but did not reach conventional significance. Strictly speaking, then, *Hypothesis 2a* received weak support. Even so, the direction of the coefficients is theoretically meaningful and broadly consistent with scholarship on invisible labour and institutional recognition. One reason may be that legibility does not fully eliminate strain, but instead weakens the broader stress process when coping efforts are recognised and converted into support or career value. Research has shown that mentoring, pastoral care, and relational co-ordination can be protective when they are recognised and rewarded, but depleting when they are treated as naturalised women’s work (Acker, 2012; Husu, 2022). This pattern is also consistent with Quickfall (2025), who shows that women in English universities undertake substantial emotional and mentoring labour that remains undervalued in advancement systems. At the same time, Gedro et al. (2020) found that mentoring opportunities did not necessarily improve retention among Black women administrators, and Rauhaus and Carr (2020) showed that advising and mentoring burdens could reduce women’s leadership progression. These studies reinforce an important point central to this paper: coping activity is not inherently buffering. Its value depends on whether it is institutionally legible and convertible into capital.

*Hypothesis 2b* predicted that the sequential indirect effect of role stress on leadership avoidance via personal strain and leader identity threat would be stronger when coping-capital legibility was low and weaker when it was high. This hypothesis received better support than H2a. The conditional indirect estimates showed a clear attenuation pattern, especially in the UK and, to a lesser extent, in Pakistan. This suggests that coping-capital legibility may matter more for the unfolding of the full process than for the isolated first-stage interaction alone. In other words, when coping work is more legible, the stress to strain to threat to avoidance pathway weakens. This supports the idea that institutional recognition matters not only for well-being but also for leadership trajectories. The weaker evidence in Pakistan may reflect limited institutional infrastructure for recognising coping work, which makes variation in legibility smaller or less actionable.

*Hypothesis 3a* proposed that role-congruity climate would strengthen the relationship between leader identity threat and leadership avoidance. This hypothesis was supported in the UK, but not clearly supported in Pakistan. The UK finding is consistent with role-congruity research showing that women face stronger backlash when leadership norms remain masculinised despite formal equality frameworks (Howe-Walsh and Turnbull, 2016; Quickfall, 2025). This may have emerged because identity threat is more likely to turn into withdrawal where women anticipate social penalties for authority. The weaker Pakistan result may appear counterintuitive given the stronger patriarchal norms documented in that context (Bhatti and Ali, 2021). However, a plausible explanation is that role-congruity pressure is already so embedded in that setting that it operates more as a background condition than as a variable moderator. Contradictory evidence also exists. Qadhi et al. (2024) found that women in male-dominated academic settings could still form workable leadership identities rather than moving directly towards withdrawal. This again suggests that identity threat does not operate uniformly and may be shaped by local identity work, role models, and informal support.

*Hypothesis 3b* predicted that the sequential indirect effect of role stress on leadership avoidance via personal strain and leader identity threat would be stronger under high role-congruity climate. This hypothesis was supported more clearly in the UK than in Pakistan, though the Pakistan pattern moved in the expected direction. The UK result reinforces the broader interpretation of the study: leadership avoidance is most likely when demands generate depletion and when the leadership context makes authority identity-risking for women. This may be because a stronger role-congruity climate increases the likelihood that identity threat translates into a behavioural decision to withdraw. The weaker moderation in Pakistan suggests that the climate effect may be less about variation in gendered norms and more about the general salience of structural barriers. Put differently, in the UK, role-congruity climate appears to amplify an already active process. In Pakistan, the same climate may be more constant, which reduces its observed moderating power.

To conclude, the findings suggest that leadership avoidance among women academics is best understood as a contextually rational response to demanding and gendered institutional environments rather than as a simple deficit of aspiration. Across both countries, role stress increased avoidance directly and indirectly through strain and identity threat, but the full integration of these pathways was stronger in the UK than in Pakistan. This suggests that the same broad mechanism may operate differently depending on whether institutional pressures are subtle and managerial or more overt and structurally entrenched. The results also show that institutional recognition matters: where coping work is more legible and where leadership climates are less identity-risking, the pathway to avoidance weakens.

### 5.1 Theoretical implications

This study advances theory by showing that institutional context shapes not only the level of stress women academics experience, but also whether that stress becomes identity-threatening and translates into leadership avoidance. The shift in debate from national culture alone to the institutional conditions that govern visibility, evaluation, and recognition goes beyond cultural essentialism and towards institutional contingency (Crenshaw, 1989; Shah, 2015).

A second contribution is to refine role stress and occupational stress perspectives by showing that similar demands at work do not produce uniform psychological outcomes across contexts. Instead, institutional arrangements influence whether strain intensifies, whether leadership becomes identity-risky, and whether avoidance becomes a rational response. This extends existing work on managerialism, gendered leadership norms, and academic labour by locating women’s leadership avoidance within meso-level organisational systems rather than treating it as an individual disposition (Gill, 2009; Morley, 2013; O’Connor, 2014). It also aligns with recent evidence that women’s leadership barriers are enacted differently across higher education systems through service invisibility, evaluative penalties, and gendered expectations (Avolio et al., 2024; Meza-Mejia et al., 2023; Yahya et al., 2024).

A third contribution is the distinction between coping capacity and coping-capital legibility. Existing stress models typically treat coping as a personal resource. This study shows that coping is only protective when institutions recognise and reward it. Where mentoring, relational co-ordination, and emotional labour remain invisible, coping may cease to restore resources and instead become depleting. The concept of coping-capital legibility therefore extends the OSI-R framework by introducing an institutional recognition lens that explains why similar coping efforts can produce different career and psychological consequences across settings (Acker, 2012; Guarino and Borden, 2017; Harris et al., 2024).

This study reframes coping from an individual trait to an exchange process. Coping behaviours are not inherently resource-generative; they become resource-generative when the institution renders them visible and tradable. That shift in emphasis aligns COR logic with organisational recognition systems and suggests that “resource caravans” in academic work are partly governed by promotion and workload infrastructures, not solely by personal coping capacity.

From developed to developing systems context, similar structural forces persist. Societal expectations, service-heavy workloads, and opaque promotion systems erode resources for visible leadership engagement (Alghamdi, 2017). This loss, combined with heightened identity threat, leads to strategic avoidance as a self-preserving response (BlackChen, 2015; O’Connor, 2014). On the other hand, in terms of mechanism level, three key research strands converge. First, occupational stress research shows that role overload and ambiguity lead to psychological strain and avoidance when support, recognition, or autonomy are limited (Colligan and Higgins, 2006; Le Fevre et al., 2006). Second, gender and leadership scholarship highlight how role-congruity pressures delegitimize women’s leadership expressions, exacerbating identity threats (Eagly and Carli, 2007; Carnes et al., 2015). Third, organisational recognition research reveals that coping efforts, such as mentoring or emotional labor, remain invisible in performance systems, preventing the replenishment of social or symbolic capital (Acker, 2012; Husu, 2022).

### 5.2 Practical and policy implications

Practically, this study suggests that coping-capital recognition functions as a buffering capability in the operating model. It should be treated as conditional evidence aligned to the indirect-effect table, not as a definitively confirmed interaction effect, and role-congruity pressures amplify the translation of identity threat into leadership avoidance, with the clearest confirmatory evidence in the UK interaction term and supportive conditional indirect patterns in both contexts. From managerial perspective, if institutions want to reduce leadership avoidance, they need to change the incentive and recognition architecture. The lever is coping-capital legibility: make coping labour visible, creditable, and exchangeable for workload relief, promotion capital, and leadership legitimacy. Where role-congruity climates remain high, leadership roles will continue to look like asymmetric bets, and women will keep opting out as a rational decision.

Universities can reduce leadership avoidance only if they change the organisational economics that make leadership feel high-risk and low-return for women. Our model points to three leverage points that are institutionally controllable: how workload and contribution are priced, what “good leadership” looks like in local evaluation, and whether identity resources are built through credible sponsorship rather than informal encouragement.

First, make coping labour measurable and promotable. The UK pattern is consistent with a scenario where mentoring, pastoral care, and boundary-spanning service stabilise departments but do not reliably convert into workload relief or progression credit. The fix is not another wellbeing intervention. It is a redesign of workload allocation and promotion criteria so that these contributions are recorded, weighted, and audited, with clear thresholds that translate into time, recognition, or advancement. A practical starting point is an annual service and mentoring ledger at school level, paired with promotion outcomes review that test whether high service loads depress leadership participation and progression.

### 5.3 Limitations and future research

This study has limitations that should be treated as boundary conditions on inference. First, the cross-sectional design constrains causal and temporal claims. Although the proposed mediation and moderation structure is theory-driven and supported by robustness checks, cross-sectional SEM cannot confirm that role stress precedes strain, that strain precedes leader identity threat, or that these dynamics are reversible. Reciprocal relationships remain plausible, for example leadership avoidance may reduce exposure to leadership-related threat while simultaneously shaping perceived strain through role selection. Future work should therefore use time-lagged, longitudinal, experience-sampling, or quasi-experimental designs to test temporal sequencing and reversibility more directly.

Second, generalisability is limited by the sampling frame, which focused on public-sector universities. Public institutions often differ from private or specialised providers in governance arrangements, performance management intensity, and resource allocation. These differences can affect leadership incentives, workload models, and the visibility and valuation of service and relational labour, potentially shifting the strength of the stress–strain–threat–avoidance pathway and its boundary conditions. Replication in private universities and more marketised systems is needed.

Third, we did not collect detailed family-structure variables (for example marital status, parental status, or joint versus nuclear household arrangements). These life-course constraints can shape leadership trade-offs and may be particularly salient where caregiving intensity is high. Future research should incorporate these factors as moderators and explore intersectional contingencies such as career stage, tenure security, and caregiving status.

Fourth, leadership avoidance should not be reduced to an exclusively adaptive response to structural barriers. Individual differences in motives and agency, including leadership identity construction, career aspirations, need for autonomy, and perceived growth opportunities, may alter whether individuals persist or withdraw under resource depletion (DeRue and Ashford, 2010). Multi-source designs that combine survey data with workload records, HR metrics, and promotion outcomes would strengthen construct validity, reduce common-source bias, and enable evaluation of interventions aimed at workload visibility and recognition systems. Finally, although scalar invariance was supported, modest fit deterioration across invariance steps suggests caution when interpreting latent mean differences. Replications with larger and more diverse samples would improve precision and clarify how institutional climates translate occupational stress into identity-protective leadership avoidance.

## Data Availability

The datasets presented in this article are not readily available because data are owned by affiliated institutions, accessed with institutional restrictive access and authors are not permitted to share the data. Requests to access the datasets should be directed to fawadahmed1@live.com.
